# Mechanism of Activating the Proprioceptive NT-3/TrkC Signalling Pathway by Reverse Intervention for the Anterior Cruciate Ligament–Hamstring Reflex Arc with Electroacupuncture

**DOI:** 10.1155/2018/6348764

**Published:** 2018-01-18

**Authors:** Lei Zhang, Yan Zeng, Ji Qi, Taiyuan Guan, Xin Zhou, Yancheng He, Guoyou Wang, Shijie Fu

**Affiliations:** ^1^The Affiliated Traditional Chinese Medicine Hospital of Southwest Medical University, Luzhou, Sichuan 646600, China; ^2^School of Traditional Chinese Medicine, Southern Medical University, Guangzhou, Guangdong Province 510515, China

## Abstract

The anterior cruciate ligament (ACL) is an important structure maintaining stability of the knee joints. Deficits in physical stability and the proprioceptive capabilities of the knee joints are observed, when the ACL is damaged. Additionally, a unilateral ACL injury can affect bilateral knee proprioception; therefore, proprioception of the ACL may play a key role in stability. Electroacupuncture therapy has a definite effect nerve regeneration. In this study, cynomolgus monkeys were randomly divided into 4 groups: the model control group, intervention of the injured knee with electroacupuncture (IIKE) group, intervention of the bilateral knees with electroacupuncture (IBKE) group, and the blank control group. The unilateral ACL injury model was developed in IIKE and IBKE groups; acupuncture points around the knees underwent intervention similarly in the IIKE and IBKE groups. Then, mRNA and protein expressions of NT-3 and TrkC in the dorsal root ganglion and of growth-associated protein-43 in the ACL increased according to reverse-transcription quantitative polymerase chain reaction and Western blotting results. Decreased incubations and increased amplitudes were found for somatosensory-evoked potentials and motor nerve conduction velocity. The finding indicates that electroacupuncture may play an important role in the recovery of proprioception in the ACL by activating the NT-3/TrkC signalling pathway.

## 1. Introduction

The anterior cruciate ligament (ACL) is an important structure that maintains knee joint stability [1–3]. ACL tears most commonly occur in active people and athletes [4] and may cause proprioceptive degeneration, which is the main factor leading to the instability of the knee joints. Proprioception refers to when proprioceptors of muscles, tendons and joints and the sense of position, motion perception, and vibration, which mainly occur in the nerve tissue, are able to complete information transmission [5–8]. However, it is unclear whether an ACL injury in the unilateral knee had impacts on the contralateral proprioception. Therefore, how to effectively restore proprioception of the injured ACL is gaining attention.

The ACL–hamstring reflex arc is an important feedback mechanism for reflexively regulating muscular tension and coordination of the knee joints that can be expressed by proprioception [9]. When the ACL is pulled by forward displacement of the tibia, the contraction of the hamstring confronts the tibia translation through regulating the ACL–hamstring reflex arc, which protects the ACL from injury [10]. Several studies have been conducted to prove the existence of the ACL–hamstring reflex arc and proprioceptive regeneration [11–13].

Many studies have found that neurotrophin-3 (NT-3), one of the neurotrophic factors, and its specific receptor tyrosine protein kinase C (TrkC) play important roles in the neural transmission of proprioception, proprioceptive rehabilitation, and nerve regeneration [14–18]. Proprioceptive neurons located in the spinal dorsal root ganglion (DRG) express TrkC, suggesting that NT-3 impacts the proprioceptive neurons [19]. Growth associated protein-43 (GAP-43) is a specific protein that widely exists in the nervous system [20] and is highly expressed in neuronal axons [21, 22]. If the ACLs were injured, then the expression of NT-3 would decrease in the proprioceptors, thus reducing the activity of TrkC; however, GAP-43 in the ACL would be activated, thereby starting the self-healing system.

Previous studies [23, 24] have shown that electricity used to stimulate ACL could reflexively activate the related flexor muscles or extensor muscles participating in stabilising activities of the knee joints. When knee joint stability is strengthened, proprioception could be well recovered, preventing reinjury of the ACL [25, 26].

Clinically, electroacupuncture therapy for acupuncture points around the knee joints is efficient for the treatment and recovery of local injuries. However, thus far, no relevant research has definitively clarified the treatment mechanism of electroacupuncture, especially for improving the proprioception function. Therefore, this study aimed to explore the mechanism of activating the proprioceptive NT-3/TrkC signalling pathway by reverse intervention for the ACL–hamstring reflex arc with electroacupuncture.

## 2. Materials and Methods

### 2.1. Ethical Statement

All procedures were approved by the Ethical Inspection Committee of Animal Experiments of Yunnan Yinmore Biological Technology Co. Ltd. (number 2016001). Animal cares were performed in accordance with the* Guide for the Care and Use of Laboratory Animals* (Office of Science and Health Reports CPRR/NIH 1996). And animal research facilities were in accordance with the Association for Assessment and Accreditation of Laboratory Animal Care International (AAALAC).

### 2.2. Experimental Animals and Feeding Procedures

Thirty male, specific pathogen-free (SPF) cynomolgus monkeys were bred for the purpose of this study and purchased from Yunnan Yinmore Biological Technology Co. Ltd. (age range, 4.0 to 5.0 years; weight range, 6.0 to 7.0 kg). All monkeys were housed in several stable cages during periods of sleeping, activities, feeding, and resting at the Laboratory Animals Breeding Center of Yunnan Yinmore Biological Technology Co. Ltd. (SYXK2009-0003).

### 2.3. Grouping and Modelling

Thirty cynomolgus monkeys were randomly divided into 4 groups: blank control group (*n* = 3); model control group (*n* = 9); intervention for the injured knee with electroacupuncture (IIKE) group (*n* = 9); and intervention for the bilateral knees with electroacupuncture (IBKE) group (*n* = 9). In the model control, IIKE, and IBKE groups, the unilateral ACL injury mode was developed using arthroscopy.

Arthroscopic instruments (Smith & Nephew, Memphis, TN, USA) were prepared before the procedure and the equipment was rigorously sterilised by operators. Under anaesthesia (5 mg/kg intramuscularly [IM]; Zoletil 50; Virbac, Carros, France), the monkeys were placed in the supine position and the surgical area was shaved. Then, incisions were marked in order with a tourniquet (ZSGB-BIO, Beijing, China). Small incisions were made around the knee joints where the scope and surgical instruments would be inserted. After preparations were completed, anterior medial and anterior lateral approaches to the knee joints were performed using 0.5 cm long incisions to explore the joints. The scope was inserted in the knee joints. Saline solution was administered through a tube and into the knees to expand the joints and improve visualization. The image was sent to a video monitor so that the surgeon could see inside the joints. Meticulous diagnostic arthroscopy was performed to evaluate the knee structures, including the meniscus, articular cartilage, ACL, and posterior cruciate ligament (PCL). Approximately one-fourth of the ACL was transversely cut using arthroscopy. At the end of the procedure, the surgical instruments were removed, and the skin incisions were closed with sutures (ZSGB-BIO). A bandage was wrapped around the knee joints and the monkey was taken to recovery. Modelling procedures were completed by the same surgeons. Soft padded bandages were placed and maintained on the operated limbs for 1 week. On postoperative day 3, an injection of levofloxacin hydrochloride and sodium chloride (8 mg/kg intravenously, once every 12 hours; Heng Ao, Zhejiang, China) was used to prevent infection. Moreover, monkeys were monitored daily and tramadol hydrochloride was injected (2 mg/kg, once/day, IM; QiMaiTe, China) to relieve pain as needed. The incisions healed within 7 days.

### 2.4. Electroacupuncture Intervention

In the IIKE and IBKE groups, monkeys were subjected to intervention with electroacupuncture at 7 days postoperatively. Acupuncture points were selected, including Wei Yang (BL39), Yin Gu (KI10), Xi Yangguan (GB33), and Qu Quan (LR8), which are all located on the hamstring.

While awake and after routine disinfection, filiform needles (Zhongyan Taihe, Beijing, China) that were 0.3 mm in diameter and 1.5 inches in length were slightly tilted inward to a depth of approximately 0.5 to 0.8 inches. Monkeys were subjected to proper lifting, thrusting, and rotating techniques to induce muscle contraction of the hamstring. After insertion in the acupuncture points for approximately 15 minutes, the needles were connected to stimulator electrodes. The stimulation frequency was fixed at 2/100 Hz with alternating dispersed and dense waves; a current intensity of 3 mA was selected. Electroacupuncture treatment was performed for 10 minutes each time. Intervention was performed every day for 4 weeks. The entire process was performed by the same acupuncturist.

**Figure 1:**
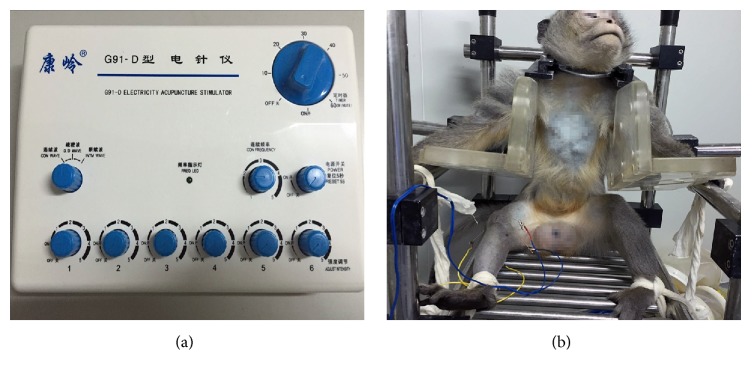
*Cynomolgus monkeys underwent electroacupuncture intervention while awake*. (a) The acupuncture point nerve stimulator (G91-D; Kangling, Zhejiang, China, G91-D). (b) A cynomolgus monkeys was placed on the fixture and subjected to electroacupuncture.

The blank control and model control groups did not undergo any intervention. In the IIKE group, electroacupuncture intervention for the injured knee was applied; the same intervention was applied for the bilateral knees (the injured knee and healthy knee) in the IBKE group (Figure 1).

At 4, 8, and 12 weeks after electroacupuncture intervention, 3 monkeys were separately and randomly chosen from the model control group, IIKE group, and IBKE group, and the proprioception changes of the ACL were examined using the neural electrophysiological method (somatosensory-evoked potentials [SEPs] and motor nerve conduction velocity [MCV]), reverse-transcription quantitative polymerase chain reaction (RT-qPCR), and Western blotting. SEPs and MCV are currently used for monitoring of peripheral nerve injuries; their indicators are incubations and amplitudes. Extended incubations and decreased amplitudes over time indicated nerve injury and proprioceptive degeneration. RT-qPCR and Western blotting were used to examine mRNA and protein expressions of NT-3, TrkC, and GAP-43. Examinations were performed for monkeys in the blank control group after group division.

### 2.5. SEPs

**Figure 2:**
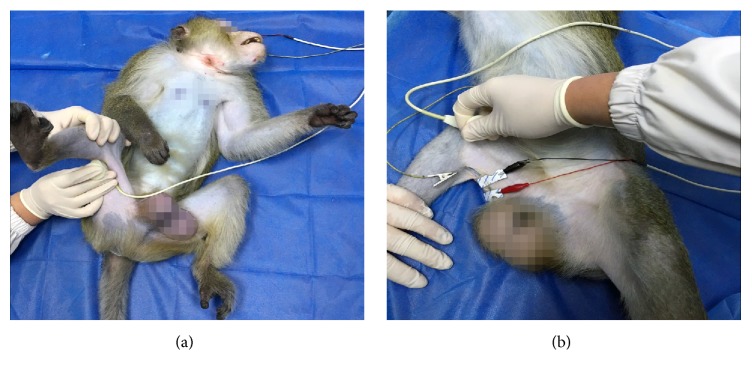
*Cynomolgus monkeys underwent electrophysiological examination involving somatosensory-evoked potentials (SEPs) and motor nerve conduction velocity (MCV) under general anaesthesia*. (a) SEPs examination. (b) MCV examination.

Monkeys were given Zoletil 50 (5 mg/kg IM) for general anaesthesia; after anaesthesia, the heads and limbs of the monkeys were fixed and only the injured knee was examined. Electrode points were 1 cm from the point of intersection of the bilateral ear tips attachments, the root of the nose, and the inion (lower limb cortex area); the reference electrode point was placed in the nasal root, and the ground wires were connected to the ears. SEPs were measured at 26–28°C, and the bipolar surface electrodes were used to stimulate the area of the body corresponding to both sides of the ACL. Stimulation parameters were constant voltage, and electrical stimulations of a single square wave were used (wave width, 0.1 ms; frequency, 2 Hz; stimulation intensity range, 15 to 20 mA). Incubations and amplitudes of SEPs for cynomolgus monkeys were recorded by the evoked potentiometer (MEB-9402C; Nihon kohden, Tokyo, Japan). Eventually, the information was entered in the microcomputer operation system, and the graphics, incubations, and amplitudes of SEPs were measured and analysed (Figure 2(a)).

### 2.6. MCV

The stimulation electrode was placed in the popliteal space for stimulation, the recording electrode was placed on the muscle belly of the hamstring at a temperature of 26–28°C, the reference electrode was placed 2 cm from the recording electrode, and bipolar surface electrodes were placed on the ACL for stimulation. Stimulation parameters were constant voltage, wave width of 0.2 ms, frequency of 1 Hz, and stimulation intensity range of 25 to 30 mA. Then, MCV incubations and amplitudes were recorded by the evoked potentiometer. Finally, the information was entered in the microcomputer operation system, and the MCV graphics, incubations, and amplitudes were measured and analysed (Figure 2(b)). Only the injured knee was examined.

### 2.7. Specimen Extraction

**Figure 3:**
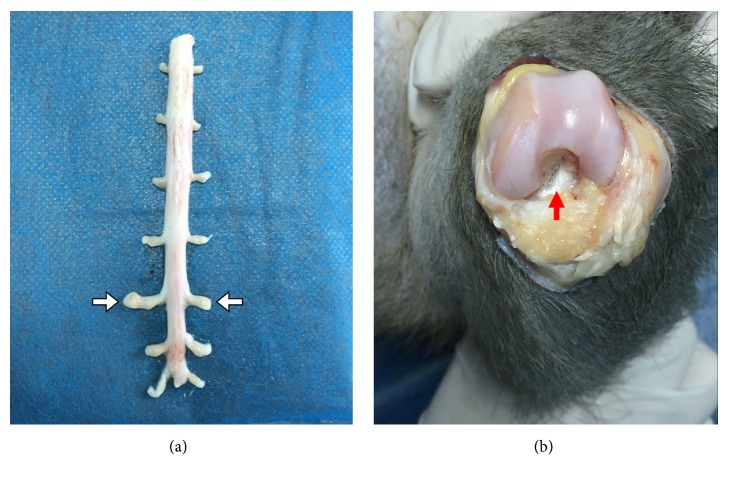
*Obtaining experimental specimens*. (a) The white arrows indicate the dorsal root ganglion (DRG). (b) The red arrow indicates the anterior cruciate ligament (ACL). Bilateral DRGs were obtained from all groups. Bilateral ACLs were obtained from the blank control group. Injured ACLs were obtained from the model control, IIKE, and IBKE groups.

Monkeys were euthanised with Zoletil 50, and the DRG and ACL were rapidly exposed and taken out to be used as experimental materials (Figure 3). They were washed with physiological saline (ZSGB-BIO) without RNase destroyer (ABI, Vernon, CA, USA), placed in 5 ml frozen pipes (Sangon, Shanghai, China), quickly plunged into liquid nitrogen (ABI), and kept in the refrigerator at −80°C. Bilateral DRGs were obtained from the 4 groups, bilateral ACLs were obtained from the blank control group, and injured ACLs were obtained from the model control, IIKE, and IBKE groups.

### 2.8. RT-qPCR

Total RNA was isolated from the DRG using TRIZOL reagent (Sangon). The concentration and purity of the extracted RNA were determined by ultramicronucleic acid protein detector (AlphaSpec; Alpha Innotech, San Leandro, CA, USA). The primers of the target gene and reference gene were provided by Sangon and designed and synthesised using Primer Premier 5.0 software. Reference gene (*β*-actin) primer sequences were R-*β*-actin forward: 5′-GATCAAGATCATTGCTCCTCCTG-3′, 58.93; and R-*β*-actin reverse: 5′-GTCACAGTCCGCCTAGAAGC-3′, 60.46, 163 bp. Target gene primer sequences were R-NT-3 forward: 5′-TGCCACGATCTTACAGGTGAAC-3′, 60.61; R-NT-3 reverse: 5′-TCCTTAACGTCCACCATCTGC-3′, 60.07, 198 bp; R-TrkC forward: 5′-CAAATGCTCCACATCGCCAG-3′, 59.9; R-TrkC reverse: 5′-CCTCCCACCCTGTAATAATCCG-3′, 59.96, 179 bp; R-GAP-43 forward: 5′-TCCACTGATAACTCGCCGTC-3′, 59.55; and R-GAP-43 reverse: 5′-TCCACTGATAACTCGCCGTC-3′, 60.44, 98 bp. cDNA was generated by total RNA using a reverse-transcriptase cDNA synthesis kit. With SYBR Green (SYBR Premix Ex Taq II; Axygen, Union City, CA, USA) as the fluorescent label and *β*-actin as the internal control, 1 *μ*l cDNA was added to the 20 *μ*l reaction system for 40 cycles for amplification. Data results were analysed using the relative quantitative method of 2^−ΔΔCt^.

### 2.9. Western Blotting

After grinding with liquid nitrogen, each 0.3 g sample was added to 1 ml protein extraction pyrolysis liquid (Sangon) and placed in an ice box (PCR Cooler; Eppendorf, Hamburg, Germany) for ultrasonic crushing. Then, the centrifuged supernatant fluid (Multifuge X3; Thermo Fisher Scientific, Waltham, MA, USA) was determined quantitatively using the bicinchoninic acid assay. After the concentration was adjusted, 20 *μ*l protein sample buffer was added to 20 *μ*l sample and boiled for 10 minutes. The pretreated protein samples were separated by SDS-PAGE and transferred to PVDF membranes (Sangon). Then, the PVDF membranes were moved to TBST solution (Sangon) for blocking. The membrane was incubated with the primary antibody solution for 1 to 2 hours at 20°C on a shaking table. After being washed with TBST solution repeatedly, the membrane was incubated for 1 to 2 hours at 20°C with the conjugated antibody (Abcam, Cambridge, MA, USA). The bands were visualized using ECL (Sangon) and the pictures were conserved by a chemiluminescence imaging apparatus (SmartChemi 610; Sage, Beijing, China).

### 2.10. Statistical Analysis

Measurement data were expressed as mean ± SD. RT-qPCR was performed during three separate experiments and data were collected. Statistical differences between the 4 groups were assessed by one-way analysis of variance (ANOVA) followed by the least significant difference (LSD) *t*-test. In the same group, differences in the repeated-measurement ANOVA and LSD *t*-test results were compared among three different time points. All statistical analyses were performed using SPSS 20.0 software (IBM Corp., Armonk, NY, USA). *P* < 0.05 was considered statistically significant.

## 3. Results

### 3.1. Neural Electrophysiological Indices

At 4, 8, and 12 weeks, compared with the blank control group, the incubations of SEPs and MCV in the model control, IIKE, and IBKE groups were extended, but the amplitudes of SEPs and MCV were decreased. The changes of the IBKE group were better than those of the IIKE group, and the changes of the IIKE group were better than those of the model control group. All differences were statistically significant (*P* < 0.05).

**Figure 4:**
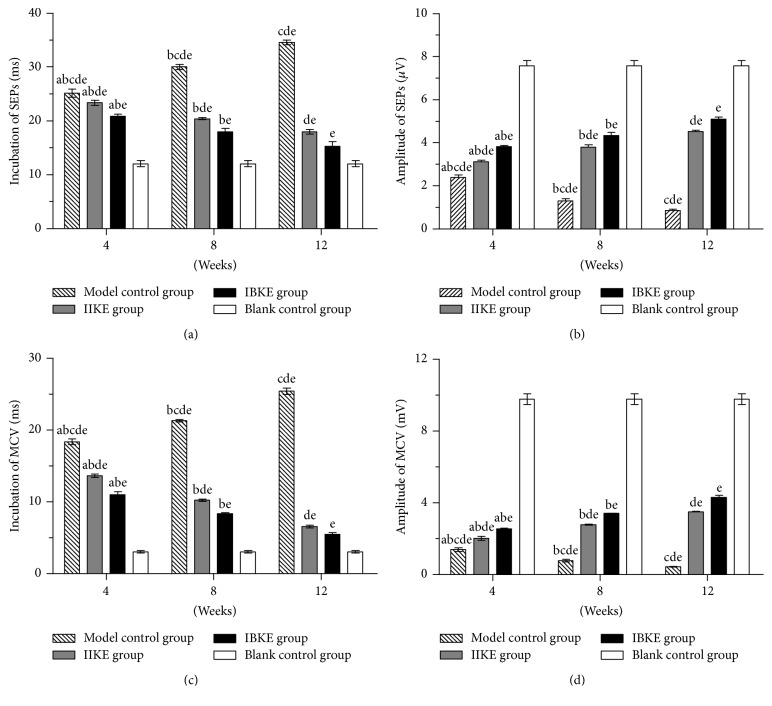
*Comparison of incubations and amplitudes of SEPs and MCV among all groups at different time points*. (a) Incubations of SEPs. (b) Amplitudes of SEPs. (c) Incubations of MCV. (d) Amplitudes of MCV. a: versus 8 weeks in the same group, *P* < 0.05. b: versus 12 weeks in the same group, *P* < 0.05. c: versus the IIKE group at the same time, *P* < 0.05. d: versus the IBKE group at the same time, *P* < 0.05; e: versus the blank control group, *P* < 0.05.

In addition, in the model control group, the incubations of the SEPs and MCV were extended over time, but the amplitudes of SEPs and MCV were decreased. In the IIKE and the IBKE groups, the incubations of SEPs and MCV were decreased, but the amplitudes of SEPs and MCV were extended. Differences were statistically significant (*P* < 0.05) (Figure 4).

### 3.2. RT-qPCR and Western Blotting

At the same time point, regarding DRGs, when comparing the mRNA and protein expressions of NT-3 and TrkC, fewer changes occurred in the model control, IIKE, and IIKE groups than in the blank control group. However, more changes occurred in the IBKE group than in the IIKE group. More changes also occurred in the IIKE group than in the model control group. All differences were statistically significant (*P* < 0.05). Regarding the ACL, when comparing the mRNA and protein expressions of GAP-43, more changes occurred in the model control group, IIKE group, and IBKE group than in the blank control group, more changes occurred in the IBKE group than in the IIKE group, and more changes occurred in the IIKE group than in the model control group. All changes were statistically significant (*P* < 0.05).

**Figure 5:**
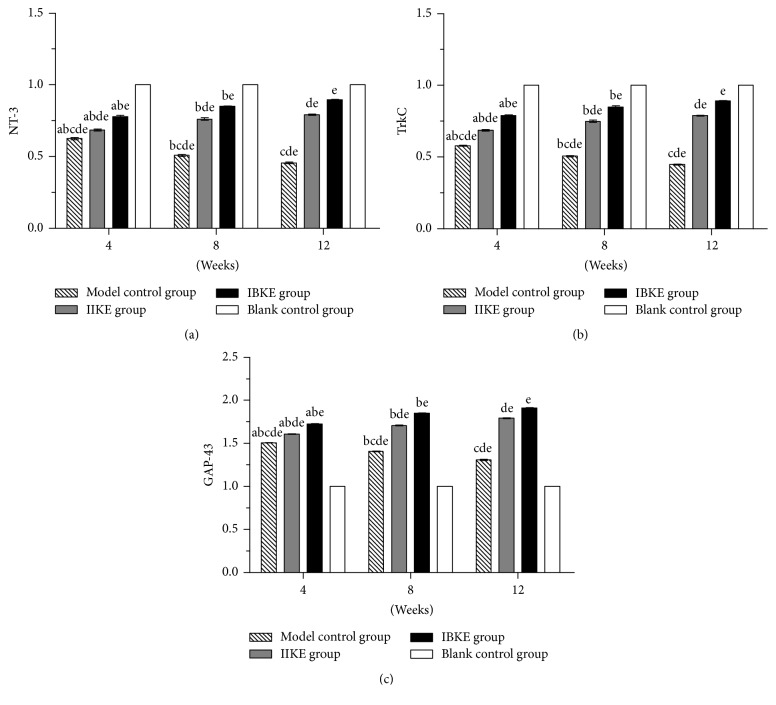
*Expressions of mRNA of NT-3, TrkC, and GAP-43 among all groups at different time points*. (a) Expression of NT-3 mRNA. (b) Expression of TrkC mRNA. (c) Expression of GAP-43 mRNA. a: versus 8 weeks in the same group, *P* < 0.05. b: versus 12 weeks in the same group, *P* < 0.05. c: versus the IIKE group at the same time, *P* < 0.05. d: versus the IBKE group at the same time, *P* < 0.05. e: versus the blank control group, *P* < 0.05.

**Figure 6:**
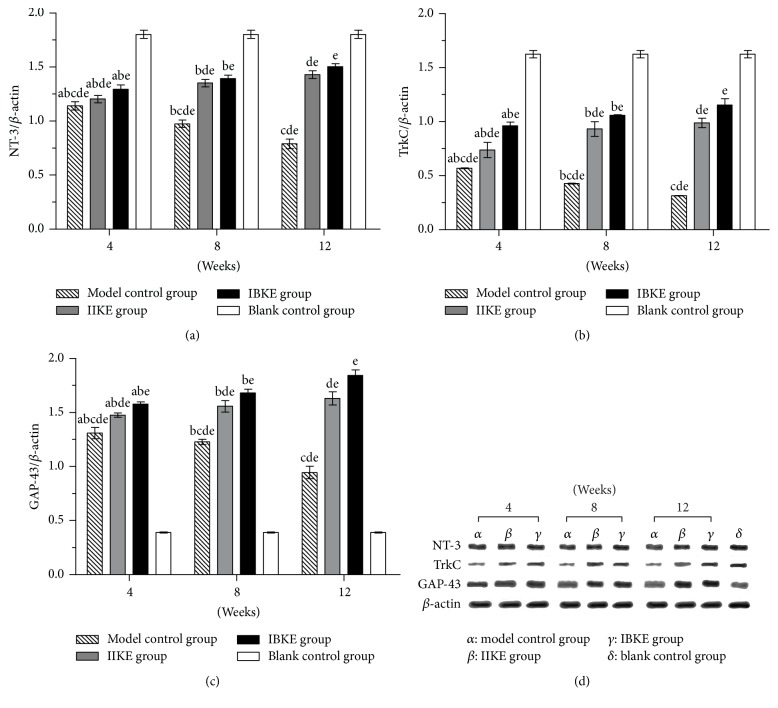
*Expressions of NT-3/β-actin, TrkC/β-actin, and GAP-43/β-actin proteins among the four groups at different time points*. (a) Expressions of NT-3/*β*-actin proteins. (b) Expressions of TrkC/*β*-actin proteins. (c) Expressions of GAP-43/*β*-actin proteins. a: versus 8 weeks in the same group, *P* < 0.05. b: versus 12 weeks in the same group, *P* < 0.05. c: versus the IIKE group at the same time, *P* < 0.05. d: versus the IBKE group at the same time, *P* < 0.05. e: versus the blank control group, *P* < 0.05. (d) The expression of protein of NT-3, TrkC, and *β*-actin among the four groups in different time points.

At different time points, in terms of mRNA and protein expressions of NT-3 and TrkC, there were fewer changes in the model control, IIKE, and IBKE groups than in the blank control group; however, the expressions of the IIKE and IBKE groups increased over time, and the differences were statistically significant (*P* < 0.05). In contrast, the expressions of the model control group decreased over time, and the differences were statistically significant (*P* < 0.05). In terms of the mRNA and protein expressions of GAP-43, those of the model control group decreased over time. However, the expressions of the IIKE and IBKE groups increased with increased intervention time. All changes were statistically significant (*P* < 0.05) (Figures 5 and 6).

## 4. Discussion

Proprioception of the ACL is closely related to knee joint stability, which is realised through feedback regulation of the central nervous system; however, the key node on the pathway remains unknown [27–30]. Many studies confirmed that when the ACL proprioception function is decreased, the neural feedback regulation pathway is bound to be disordered; therefore, it could have further impact on the proprioception function [31, 32]. Some studies showed that NT-3 was the key induced signal of proprioceptive transduction; when NT-3 expression was normal, the proprioceptive nerve fibres could control the target organs and have physiological roles [33–35]. If the expressions of NT-3 and TrkC were decreased, then apoptosis of proprioceptive neurons in the DRG could occur, thus causing lower limb proprioception deficiencies. NT-3 and TrkC could also accelerate the development and maturation of neurons, thus maintaining the survival of mature neurons and promoting the repair and regeneration of injured neurons. When the proprioceptive nerve conduction pathway is injured, proprioceptive neurons appear abnormal; however, exogenous NT-3 could protect the proprioceptive neurons and improve their function [36, 37]. ACL injuries could damage peripheral nerve fibres. When the nerve is damaged, expression of upregulation could be included and the functional relationship could be decreased immediately after reconstruction, which is considered as a marker of neuronal growth and repair [38].

During this study, at 4, 8, and 12 weeks, in comparison with the blank control group, incubations of SEPs and MCV in the model control group were significantly extended, but the amplitudes were significantly decreased (*P* < 0.05). Comparisons of the three time points showed that incubations of the SEPs and MCV in the model control group were significantly extended, but the amplitudes were significantly decreased over time (*P* < 0.05). Furthermore, at the same time point, in the DRG, when comparing the relative mRNA and protein expressions of NT-3 and TrkC, fewer changes occurred in the model control group than in the blank control group. In the ACL, when comparing the relative mRNA and protein expressions of GAP-43, more changes occurred in the model control group than in the blank control group; all the differences were statistically significant (*P* < 0.05). In the model control group, at different time points, the relative expression of mRNA and protein of NT-3 and TrkC decreased over time, while the relative mRNA and protein expressions of GAP-43 increased over time; all changes were statistically significant (*P* < 0.05). The results of this study illustrated that when the ACL was injured, the expressions of NT-3 and TrkC in neuronal cells of the DRG could be reduced and the levels of phosphorylation of TrkC decreased after activation, thereby further reducing TrkC activity. When the ACL was injured, GAP-43 was activated in the proprioceptors, which caused the increased expression of GAP-43. Eventually, changes in these cytokine expression levels can lead to decreased proprioceptive functions.

Electroacupuncture is a form of acupuncture whereby a small electric current is passed between pairs of acupuncture needles. The use of electroacupuncture therapy (the combination of nerve electrical stimulation and acupuncture) has gradually replaced other acupuncture therapies. Electroacupuncture therapy has been widely used to treat pain or injury in the hind limbs for many decades in China. Both human and animal experiments have confirmed that electroacupuncture therapy has a definite effect on the treatment of pain, movement disturbance, tissue regeneration, and so on [39–44]. Some studies have found that the effects of acupuncture were mediated by the central nervous system, thus causing specific physiological effect that achieved the purpose of treatment and improved the growth of neurons, axonal regeneration, synaptic reconstruction, and the functional connection of target organs [45, 46].

When the ACL is pulled by the forward displacement of the tibia, the contraction of the hamstring confronts tibial translation through regulating the ACL–hamstring reflex arc, which plays an important role in maintaining the stability of the knee joints. The acupuncture points included in this study were Wei Yang, Yin Gu, Xi Yangguan, and Qu Quan. These points were chosen because they are all located next to the hamstring. When these acupuncture points were stimulated by electroacupuncture, muscle contraction of the hamstring occurred and created the effect of reverse intervention. Furthermore, the ACL belongs to the arthromyodynia category of Traditional Chinese Medicine. Among these selected acupuncture points, Wei Yang (BL19) is the Xiahe triple energiser acupuncture point that is mainly used to treat strong pain in the lumbar spine and pain and spasms in the legs and feet. Yin Gu (KI10) is mainly used to treat strong pain in the knee joints. Xi Yangguan (GB33) is mainly used to treat swelling, pain, spasms in the knee joint and popliteal area, numbness in the leg, and other diseases of the lower limbs and knee joints. Qu Quan (LR8) is mainly used to treat swelling, pain in the knee joints and patella, and paralysis of lower limb. Through the stimulation of related points, electroacupuncture transmits a large number of proprioceptive and skin sensorial information to the central nervous system, thereby participating in stabilising the activities of the knee joints and reconstruction of the proprioceptive function of the knee joints.

In this study, at 4, 8, and 12 weeks, compared with the blank control group, SEP and MCV incubations in the model control group and the IIKE group were extended, but the amplitudes were decreased. The changes in the IIKE group were better than those in the model control group; all differences were statistically significant (*P* < 0.05). At the same time point, in the DRG, when comparing the relative mRNA and protein expressions of NT-3 and TrkC, there were fewer changes in the model control group than in the blank control group; however, more changes occurred in the IIKE group than in the model control group, in which all differences were statistically significant (*P* < 0.05). It was also indicated that electroacupuncture could reversely intervene in the ACL–hamstring reflex arc, thereby activating the NT-3/TrkC signal pathway and increasing the expression of NT-3 in the proprioceptors of hamstring. NT-3 was reversely transported to neurons in the DRG and then combined with the corresponding TrkC receptor, which promoted phosphorylation of cytosolic TrkC after receptor activation, which could enhance TrkC activity. It could also activate GAP-43 in the ACL, which enhances the polymerisation ability of GAP-43 in proprioceptors of ACL, thereby increasing the activity of ACL proprioception. The results found during our experiment may supplement certain potential explanations about the mechanisms of acupuncture treatment for ACL injury, to some extent.

Some studies have found that unilateral ACL injury could lead to proprioceptive degeneration of the bilateral knee joints [47]. In theory, because of the characteristics of nerve conduction, damage, or intervention, the proprioception of one-side limb had a negative effect on the proprioception of the contralateral limb. In this study, the IIKE group and the IBKE group were designed for the purpose of proving that intervention for the healthy side can promote the proprioceptive recovery of the injured limb through central regulation of the DRG.

In terms of nerve electrophysiological examination and the relative mRNA and protein expressions of NT-3 and TrkC in the DRG and of GAP-43 in the ACL, the recovery effect of the IBKE group was more remarkable than that of the IIKE group (*P* < 0.05). It showed that, using the DRG as the node, the proprioception of bilateral ACLs could be regulated and that the model of bilateral knee joint intervention was better than the model of unilateral knee intervention. Additionally, the effect of electroacupuncture intervention was more significant with increased intervention time (*P* < 0.05).

This research had some limitations. First, all animals were male, and it is unclear whether that affected the results. Second, because of the limitations of the objective conditions, the period of observation during the experiment was only 12 weeks; therefore, the long-term changes in proprioception could not be observed. Third, without some antagonists of the NT-3/TrkC signalling pathway, it might be insufficient to check the role of the NT-3/TrkC signalling pathway in reverse intervention for the ACL–hamstring reflex arc with electroacupuncture. Because these limitations existed, this study is considered basic. All these issues remain to be solved by further related studies.

## 5. Conclusions

In this study, the mechanism of reverse intervention for ACL proprioception with electroacupuncture, which was based on the ACL–hamstring reflex arc and through mediation of the NT-3/TrkC signalling pathway, was explored. This study helped establish the basis for the clinical application of electroacupuncture therapy and effective treatment methods and patterns for proprioception disturbances caused by ACL injuries, thereby enriching and developing the scientific connotation of electroacupuncture therapy guided by theory.
